# Variation in Ribosomal DNA in the Genus *Trifolium* (Fabaceae)

**DOI:** 10.3390/plants10091771

**Published:** 2021-08-25

**Authors:** Radka Vozárová, Eliška Macková, David Vlk, Jana Řepková

**Affiliations:** 1 Department of Experimental Biology, Faculty of Sciences, Masaryk University, 611 37 Brno, Czech Republic; vozarova.radka@gmail.com (R.V.); mackova.e.94@gmail.com (E.M.); Vlk.DavidR@email.cz (D.V.); 2 Department of Molecular Epigenetics, Institute of Biophysics, Academy of Sciences of the Czech Republic, v.v.i., Královopolská 135, 612 65 Brno, Czech Republic

**Keywords:** 5S rDNA, 26S rDNA, clover, fluorescent in situ hybridization, nucleotide polymorphism, genome structure

## Abstract

The genus *Trifolium* L. is characterized by basic chromosome numbers 8, 7, 6, and 5. We conducted a genus-wide study of ribosomal DNA (rDNA) structure variability in diploids and polyploids to gain insight into evolutionary history. We used fluorescent in situ hybridization to newly investigate rDNA variation by number and position in 30 *Trifolium* species. Evolutionary history among species was examined using 85 available sequences of internal transcribed spacer 1 (ITS1) of 35S rDNA. In diploid species with ancestral basic chromosome number (x = 8), one pair of 5S and 26S rDNA in separate or adjacent positions on a pair of chromosomes was prevalent. Genomes of species with reduced basic chromosome numbers were characterized by increased number of signals determined on one pair of chromosomes or all chromosomes. Increased number of signals was observed also in diploids *Trifolium alpestre* and *Trifolium microcephalum* and in polyploids. Sequence alignment revealed ITS1 sequences with mostly single nucleotide polymorphisms, and ITS1 diversity was greater in diploids with reduced basic chromosome numbers compared to diploids with ancestral basic chromosome number (x = 8) and polyploids. Our results suggest the presence of one 5S rDNA site and one 26S rDNA site as an ancestral state.

## 1. Introduction

A part of the third-largest plant family, Fabaceae, the genus *Trifolium* (clovers) includes ca. 255 species [1,2,3,4] with cosmopolitan distribution throughout a large range of biotopes characterized by different temperature and climate conditions. Many *Trifolium* species are extensively cultivated as fodder plants or, due to their symbiotic relationship with the nitrogen-fixing bacterium *Rhizobium leguminosarum*, as green manure crops to enhance soil fertility and sustainability [5].

The genus *Trifolium* is distributed across both the Northern and Southern hemispheres, except for Southeast Asia and Australia. More than half of its species originated in the Mediterranean region [6,7], which still has the largest number of endemic species [8,9]. The genus’ origin was estimated by Ellison et al. [3] to be in the Early Miocene (Tertiary Period), 16–23 million years ago.

Changes in chromosome number played a role in the evolution of the genus *Trifolium* [10]. While 80% of species have a basic chromosome number of x = 8, species showing reduced basic chromosome number (x = 7, 6, 5) are known [3,11]. Polyploidy has been observed in 24 species and includes tetraploidy, hexaploidy, octoploidy, dodecaploidy, and hexadecaploidy [3]. Chromosome evolution in *Trifolium* has been studied since the 1980s [1,12,13]. The first phylogenetic study in the genus was published by Ellison et al. [3]. This study is consistent with previous studies supporting x = 8 as the ancestral basic chromosome number. To date, 31 species with reduced basic chromosome numbers have been identified (x = 7, 6, 5), 11 of which exhibit both reduced and ancestral diploid or polyploid counts, while two of these species exhibit two different reduced counts [3].

*Trifolium* species have smaller to medium-sized genomes, ranging from 337.1 Mb in *Trifolium ligusticum* to 5669.3 Mb in *Trifolium pannonicum* per 1C value [14]. The full genome sequences of six *Trifolium* species are available, including of the cultured clovers *Trifolium pratense* [15,16] and *Trifolium repens* [17] and of the wild clovers *Trifolium medium* [18], *Trifolium subterraneum* [19,20], *Trifolium occidentale* [17], and *Trifolium pallescens* [17].

More detailed analyses from a phylogenetic perspective have been published based upon nuclear ribosomal DNA (rDNA) internal transcribed spacer (ITS) and chloroplast *trnL* intron sequences [3] or highly unusual *Trifolium* plastomes [21]. On the basis of chloroplast *trnL* intron sequences, phylogenetic categorization of 218 species from the *Trifolium* genus resulted in division into two subgenera: *Chronosemium* (20 species) and *Trifolium* (198 species). The latter has been further divided into eight sections: *Glycyrrhizum* (2 species), *Involucrarium* (72 species), *Lupinaster* (3 species), *Paramesus* (2 species), *Trichocephalum* (9 species), *Trifoliastrum* (20 species), *Trifolium* (73 species), and *Vesicastrum* (54 species) [3].

Ribosomal genes are among the best-known regions of genomes in eukaryotic organisms. Polycistronic gene 35S constituting the nucleolus organizer region (NOR) contains three coding genes (18S, 5.8S, and 26S) which are separated by internal transcribed spacers (ITS1 and ITS2) and an external transcribed spacer (ETS). The 5S rRNA repeat unit consists only of the coding conserved sequence 5S gene and non-transcribed intergenic spacer that separates another coding gene, 5S rRNA. Both 5S and 18S-5.8S-26S loci are located independently as tandem repeats numbering from hundreds to thousands of copies in genomes of higher vascular plants [22]. The numbers of 5S rRNA gene copies are higher than those for 35S rRNA genes, ranging from 2000 to 75,000 [23].

Due to their universal presence in eukaryotic genomes, the number, position, and structure of the 5S and 35S rDNA loci are considered important characteristics of a given species, genus, or group [24]. In angiosperms, the location of rDNA sites does not vary randomly and the distribution of sites on the chromosome arms is not the same in different taxa [25,26]. Variations in number and location of these multigene families have been used as cytogenetic markers and genomic landmarks to construct molecular cytogenetic maps [27,28], to study genome evolution [29,30,31,32], and to assess genomic relationships between closely related species [33]. Simultaneously, sequencing of internal transcribed spacers ITS1 and ITS2 and patterns of intra- and interspecific diversity have been extensively characterized and used for phylogenetic reconstruction [3,34,35].

Four nuclear rRNA genes (5S, 18S, 5.8S, and 26S) have been proven as excellent cytogenetic markers for karyotype analysis using the fluorescence in situ hybridization (FISH) technique [36]. In the genus *Trifolium*, the number and position of rDNA sites on chromosomes have been reported in seven related species (*Trifolium ambiguum*, *Trifolium hybridum*, *Trifolium isthmocarpum*, *Trifolium nigrescens*, *T. occidentale*, *T. repens*, and *Trifolium uniflorum*) belonging to the section *Trifoliastrum* [37], for two species (*Trifolium israeliticum*, *T. subterraneum* subsp. *brachycalycinum*, subsp. *subterraneum* and subsp. *yanninicum*) belonging to the section *Trichocephalum* [10], for only two species (*T. medium*, *T. pratense*) from the largest section *Trifolium* [18,27,38], and for three species (*Trifolium campestre*, *Trifolium dubium*, and *Trifolium micranthum*) from the subgenus *Chronosemium* [34]. In this study, we aim to (i) provide a comprehensive cytogenetic study of the rDNA variation with regards to the number and position of rDNA sites on chromosomes in the genus *Trifolium* as represented by 30 species (diploids and polyploids) from two subgenera and eight different sections, (ii) reconstruct the ancestral karyotype regarding number of rDNA sites, and (iii) correlate the rDNA variation with the genus-wide phylogenetic relationship using available sequences of 26S.

## 2. Results

### 2.1. rDNA Localization on Chromosomes and Range of Variation in Trifolium

**Figure 1:**
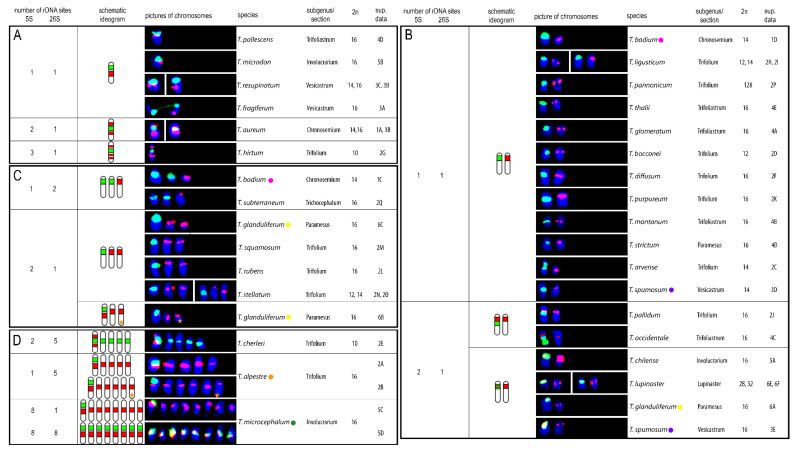
Hybridization patterns of 5S (red) and 26S (green) rDNA FISH probes in 30 *Trifolium* species. Distribution of all rDNA loci on 1 chromosome (**A**), 2 chromosomes (**B**), 3 chromosomes (**C**), and 5 and 8 chromosomes (**D**) per haploid genome was observed. A star (columns 2 and 3) indicates an odd chromosome carrying signal. A coloured circle next to a species name indicates variability in rDNA sites number and localization. Fluorescent probes were labelled with digoxigenin visualized by anti-DIG-FITC antibodies (green) or biotin visualized by streptavidin-Cy3 antibodies (red). Note that ideograms only schematically illustrate numbers of 5S and 26S rDNA loci and their mutual localization and do not precisely capture specific chromosomal localization of each locus.

Twenty-seven clover species were newly described regarding number and position of the 5S and 26S rDNA sites on chromosomes. In three additional species published previously (*T. microcephalum*, *T. occidentale*, and *T. subterraneum* subsp. *subterraneum*), different sites number or positions were observed. Hybridization patterns of 5S and 26S rDNA probes in individually analysed clover species are summarized in Figure 1, and complete DAPI karyotypes with 5S and 26S rDNA probes of all analysed species sorted according to subgenera and sections can be found in the supplementary materials (Figures S1–S6). The numbers of both 5S and 26S rDNA sites ranged from 1 to 8 per haploid genome. 5S rDNA sites were localized on 1, 2, 5, or 8 chromosomes per haploid genome (Figure 1A–D). Three species showed variability in 5S rDNA sites regarding number and localization (*T. alpestre*, *Trifolium glanduliferum*, *Trifolium spumosum*) and two species had odd chromosomes carrying signals (*T. alpestre* had 11 5S rDNA sites per diploid genome; *T. glanduliferum* had 3 5S rDNA loci per diploid genome). Similarly, 26S rDNA sites were localized on 1, 2, 5, or 8 chromosomes per haploid genome (Figure 1A–D). Variability in 26S rDNA sites number was observed in two species (*Trifolium badium*, *T. microcephalum*), and no odd chromosomes carrying signals were found.

The most common chromosomal arrangement in the analysed species was one 5S and one 26S rDNA site per haploid genome separately on two different chromosomes (Figure 1B). This constitution was observed in 12 species across subgenera *Chronosemium* (*T. badium*) and *Trifolium*, with sections *Trifolium* (*T. ligusticum*, *T. pannonicum*, *Trifolium bocconei*, *Trifolium diffusum*, *Trifolium purpureum*, and *Trifolium arvense*), *Trifoliastrum* (*Trifolium glomeratum*, *Trifolium montanum*, and *Trifolium thalii*), *Paramesus* (*Trifolium strictum*), and *Vesicastrum* (*T. spumosum*). Co-localization of one 5S and one 26S rDNA site on one chromosome was found in 12 species in total. In eight of these species another one or more chromosomes with 5S rDNA signal were described, four of which showed overlapping signals of both rDNA sites (*Trifolium chilense*, *Trifolium lupinaster*, *T. glanduliferum*, and *T. spumosum*; (Figure 1B)). In addition, for species with overlapping 5S and 26S rDNA sites, an additional one chromosome bearing 5S rDNA loci per haploid genome was observed in *Trifolium pallidum*, *T. occidentale* (Figure 1B), and *T. glanduliferum* and with one odd chromosome carrying 5S loci (Figure 1C). Further expansion of 5S rDNA loci on more chromosomes was found in *T. alpestre* (five or five with one extra odd chromosome in haploid genome) and in *T. microcephalum* (all eight chromosomes in haploid genome) (Figure 1D). Co-localization of two or three 5S rDNA signals on one chromosome was observed in *Trifolium aureum* and *Trifolium cherleri* (two 5S signals; Figure 1A,D) and *Trifolium hirtum* (three 5S signals; Figure 1A).

Expansion of 26S rDNA sites was observed more rarely. An additional one chromosome carrying 26S rDNA loci alone was observed in two species (*T. badium* and *T. subterraneum*; Figure 1C), and further expansion was found in *T. cherleri* (additional four chromosomes carrying 26S rDNA loci in haploid genome) and *T. microcephalum* (all eight chromosomes in haploid genome carrying both 5S and 26S rDNA loci) (Figure 1D).

Intraspecific variability in 5S and 26S rDNA site numbers and arrangements were described in five species. One 5S and one or two 26S rDNA sites separately on two or three chromosomes, respectively, were observed in *T. badium* (Figure 1B,C). Three arrangements were found in *T. glanduliferum*: (i) two 5S and one 26S rDNA sites separately on three chromosomes, (ii) co-localization of overlapping 5S and 26S rDNA sites on one chromosome with another chromosome carrying a 5S rDNA site, and (iii) co-localization of non-overlapping 5S and 26S rDNA sites with another one and one extra odd chromosome carrying 5S rDNA sites (Figure 1B,C). In addition to *T. glanduliferum*, an extra odd chromosome carrying 5S rDNA loci was also described in *T. alpestre* (Figure 1D). *T. microcephalum* had two variants of rDNA loci arrangement: one chromosome carrying both 5S and 26S rDNA sites with all other chromosomes carrying 5S rDNA sites or all chromosomes carrying both 5S and 26S rDNA sites (Figure 1D). Finally, in *T. spumosum*, one chromosome carrying a single 5S rDNA site was accompanied either by a chromosome carrying one 26S rDNA signal or by a chromosome having overlapping signals of both 5S and 26S rDNA loci (Figure 1B).

**Figure 2:**
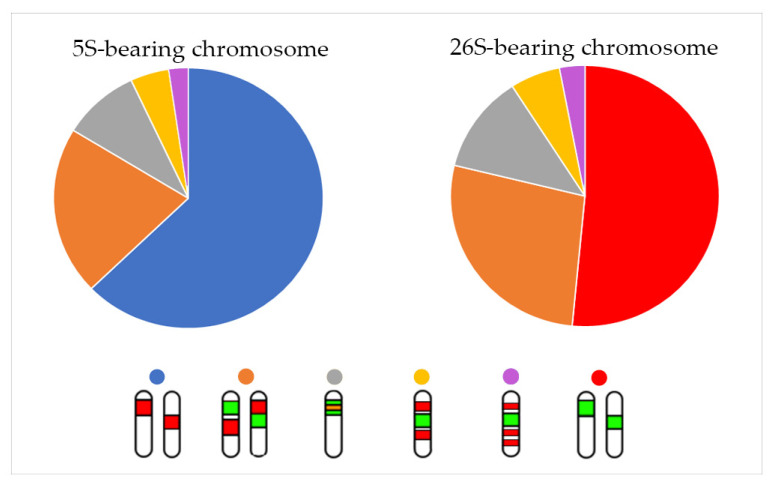
Distribution of described chromosomes bearing 5S (red) and 26S (green) rDNA sites and their arrangement in analysed species. Note that ideograms illustrate numbers of 5S and 26S rDNA loci and their mutual localizations only schematically and do not precisely capture specific chromosomal localization of each locus.

Arrangements of chromosomes bearing either 5S, 26S rDNA, or both, loci and their distributions in the analysed species are summarized in Figure 2.

Numbers and mutual localizations of rDNA sites in all species described in the present and earlier studies are summarized in Table S1. Moreover, numbers of 5S and 26S rDNA sites were further examined in species with known ITS1 sequences.

### 2.2. Reconstruction of the Ancestral Karyotype in Trifolium

**Figure 3:**
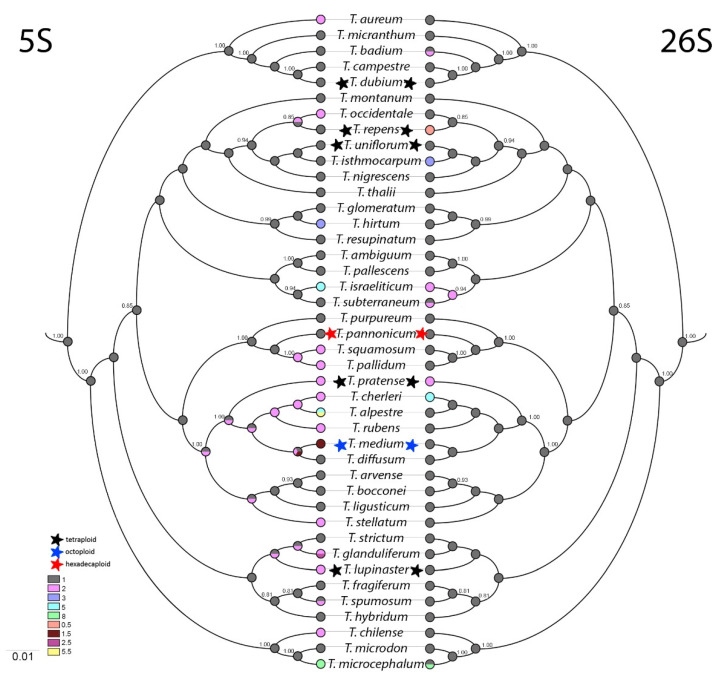
Ancestral state reconstruction of 5S (**left**) and 26S (**right**) rDNA sites numbers. ITS1 spacer of dicotyledon *A. thaliana* was chosen as the root of the tree and then omitted from the final visualization. Stars mark polyploids. Colour charts represent numbers of 5S and 26S rDNA sites per haploid genome.

Based upon ITS1 sequences, a phylogenetic tree was constructed and ancestral state reconstruction was made of 5S and 26S rDNA site numbers per haploid genome (Figure 3).

Parsimoniously estimated ancestral numbers of 5S and 26S rDNA sites suggest that one 5S and one 26S rDNA is the most likely character state for the common ancestor. Diversification of 5S rDNA numbers occurred in all analysed sections independently. The earliest event occurred in the section *Trifolium* (*T. purpureum*, *T. pannonicum*, *Trifolium squamosum*, *T. pallidum*, *T. pratense*, *T. cherleri*, *T. alpestre*, *Trifolium rubens*, *T. medium*, *T. diffusum*, *T. arvense*, *T. bocconei*, *T. ligusticum*, *Trifolium stellatum*, and *T. hirtum*). Diversification had the character of expansion from ancestral 1 to 2 or rarely to 3, 5 or 8 5S rDNA sites per haploid genome. Non-integer numbers of 5S rDNA sites per haploid genome were found in three species (in octoploid *T. medium* and in diploids *T. alpestre* and *T. glanduliferum,* both with an extra odd chromosome carrying 5S rDNA loci).

On the other hand, diversification of 26S rDNA sites was observed rarely, occurring only in subgenus *Chronosemium* and in subgenus *Trifolium* in sections *Trifolium*, *Trifoliastrum, Trichocephalum*, and *Involucrarium*. Most commonly, expansion from one to two 26S rDNA sites was observed. The only species with reduction in number of 26S rDNA sites per haploid genome was tetraploid *T. repens* (0.5 site/1 n).

### 2.3. ITS1 Sequence Variability

**Figure 4:**
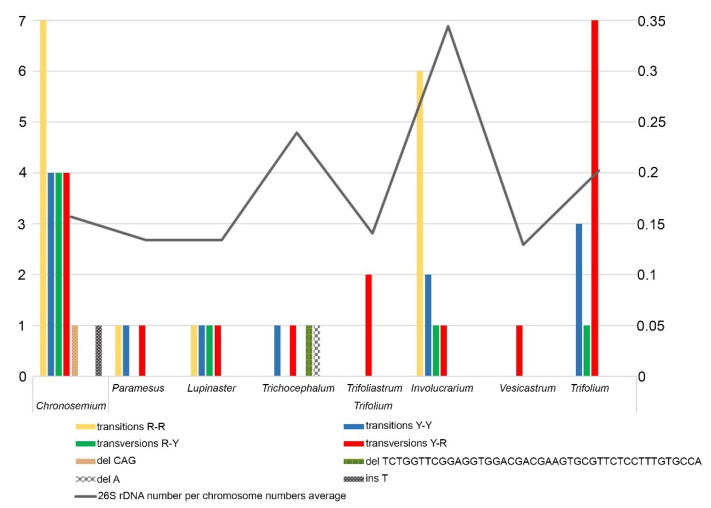
Number of substitutions, deletions, and insertions in ITS1 sequences for each *Trifolium* section (see also Table S3). Grey curve represents mean proportion of polymorphic loci per chromosome of the analysed species in individual sections.

Multiple alignment of 85 nucleotide sequences of *Trifolium* species (Table S2) resulted in identification of 56 specific polymorphisms within eight *Trifolium* sections (Table S3). These were mostly single nucleotide polymorphisms (SNPs), and only in four cases did we find indels. In accordance with this data, we created a graph showing substitutions (transitions and transversions), deletions, or insertions in each section of the two subgenera (Figure 4, Table S3). Furthermore, we show a rough correlation between polymorphisms in ITS1 sequences and mean 26S rDNA sites per chromosome in all analysed species in each section (Figure 4). The largest mean proportion of 26S rDNA sites per chromosome was observed in the section *Involucrarium*. The sections *Vesicastrum*, *Trifoliastrum*, *Paramesus*, and *Lupinaster* presented the smallest numbers of variable sites, containing just 1, 2, 3, and 4 base substitutions, respectively, and the lowest mean 26S rDNA loci proportions. These results are influenced by the amounts of input data, as not all species in the genus were analysed.

The highest number of polymorphisms (21) was identified in the section *Chronosemium*, the only section of the subgenus *Chronosemium*. These included both transitions and transversions R-Y and one species-specific indel. In subgenus *Trifolium*, the sections *Trifolium* (mainly transversions Y-R) and *Involucrarium* (mainly transitions R-R) contained the highest numbers of specific polymorphisms, at 11 and 10, respectively. Two specific deletions (one of them 43 bp long) were identified in the section *Trichocephalum*. The genetic diversity of ITS1 sequence in genus *Trifolium* is demonstrated in Table 1. To check whether the detected ITS1 sequence diversity is influenced by the presence of pseudogenes in the analysed sequences, we evaluated their presence as demonstrated by Xu et al. [35] (Table S4). GC content was analysed in three regions (ITS1, 5.8S, and ITS2), and we observed no significantly lower GC content in any region. For 5.8S rDNA sequences, all of them contained three angiosperm-conserved motifs, and each sequence was able to fold into a conserved secondary structure (Table S4). Overall, we concluded that the analysed sequences did not contain pseudogenes, and this could increase variability among analysed ITS1 regions in the *Trifolium* genus.

Estimated either by the mean number of nucleotide differences (k) or by nucleotide diversity (Pi), genetic diversity of ITS1 sequences was assessed in *Trifolium* species as divided into three groups by their ploidy and basic chromosome numbers (Table 1). The sequence diversity with respect to SNP polymorphism was lowest for diploids with ancestral basic chromosome number (ABCN) for both k and Pi, while genetic variability for diploids with reduced basic chromosome numbers (RBCN) and polyploids was comparable and higher for both values compared to diploids ABCN. Indel diversity, k(i), and indel diversity per site, Pi(i), were highest for diploids RBCN, while k(i) and Pi(i) for polyploids were lower and comparable with those for diploids ABCN. This could be a result from there being half the number of indel events (7) in polyploids compared to those in diploids RBCN and diploids ABCN, at 16 and 18, respectively.

## 3. Discussion

### 3.1. rDNA Localization on Chromosomes and Range of Variation in Trifolium

Localization of rDNA loci is highly variable. The loci occur separately on different chromosomes but also on a single chromosome pair together with varied co-localization. Analyses of rDNA loci in 100 species belonging to the family Fabaceae have been demonstrated [25]. Observations in our study showed separated positions of 5S and 26S loci on different chromosome pairs in 17 *Trifolium* species, mostly one pair of each locus or two pairs of either locus, which is very common in plants [25]. In total, co-localization of the loci 26S and 5S on a chromosome pair was observed in 16 *Trifolium* species and co-localization of 5S–5S was described in 3 species. In the plant kingdom, the most frequent co-localizations of 35S–5S (27.8%), 5S–5S (13.5%), and 35S–35S (3.4%) on a single chromosome pair was described by Roa and Guerra [26]. RNA polymerase I is used for transcription of 35S rDNA polycistronic loci, and the 5S rRNA gene is transcribed separately by RNA polymerase III [39].

Description of sectional specificities in 5S and 26S rDNA site numbers and positions could be beneficial for deducing ancestral state reconstruction in clovers. In *T. badium* (subgenus *Chronosemium*), we evaluated rDNA signals located on separated chromosomes with one or two 26S signals. A similar distribution pattern of the two types of rDNA signals was described by Ansari et al. [34] in the diploid species *T. campestre* and *T. micranthum*. In that case, a pair of 5S rDNA and a second pair of 26S rDNA signals were located on different pairs of chromosomes in both species. Separate positions of 5S and 26S rDNA sites were established to be prevalent in this section. On the other hand, a species-specific adjacent position of the 5S and 26S rDNA loci in the pericentromeric region of a pair of larger chromosomes was observed in *T. aureum*. This ordering of signals could indicate an evolutionary originally adjacent 5S and 26S rDNA position in the subgenus with possible translocation of the chromosomal material to a distal position on the chromosome arm or chromosome fusion.

The section *Trifolium* comprises species with the most diverse chromosome numbers, including both the ancestral number 2n = 16 and reduced numbers 14, 12, and 10. This diversity also relates to the manner of rDNA signal positioning (separate and adjacent) and signal numbers. Separate 5S and 26S rDNA positions were observed to prevail (in *T. arvense*, *T. bocconei*, *T. diffusum*, *T. ligusticum*, *T. purpureum*, *T. rubens*, *T. squamosum*, *T. stellatum*, and *T. pannonicum*), with 5S sites often positioned in the subterminal or terminal chromosome regions. The location of rDNA sites in the terminal chromosome region may be the result of homologous recombination constraints and chromosomal rearrangements [25], such as DNA translocations or loss.

Ancestral state reconstruction suggested one 5S and one 26S as an ancestral condition and distribution of the described chromosomes bearing 5S and 26S rDNA sites showed separate arrangements to be prevalent. *T. diffusum*, with x = 8 and a single pair of 5S and 26S separately, could represent an ancestral karyotype in the *Trifolium* section but also in the genus as a whole. Co-localization and further expansions of 5S and 26S rDNA sites could occur independently in both subgenera as a result of chromosomal rearrangements including the ancestor of subgenus *Chronosemium*.

Distinct arrangements of rDNA sites in the section *Trifolium* resembled increased number of signals of both 5S (6 in *T. hirtum*, 10 or 11 in *T. alpestre*) and 26S (10 in *T. cherleri*). While *T. cherleri* and *T. hirtum* are species with reduced basic chromosome number and 2n = 10, genomes of *T. alpestre* are characterized by the basic chromosome number x = 8. *T. cherleri* and *T. hirtum* are two examples of speciation based upon chromosomal rearrangements and reduction of chromosome number. Expansion of 5S rDNA sites in *T. alpestre*, including an odd number, shows its genome instability. Increase in 35S rDNA sites could be linked to the activation of mobile elements that can produce a transposition of rDNA copies to new genomic locations [40]. Intragenomic mobility of rRNA genes as a consequence of transposon activity has been widely reported in angiosperms, and it has been hypothesized as one of the major forces driving rDNA locus evolution [41].

Separate position of 5S and 26S rDNA sites was observed to be prevalent in the section *Trichocephalum*. One exception, as reported by Falistocco et al., was *T. israeliticum* (2n = 12) [10], which constitutes another example of speciation based upon chromosome number reduction. Those authors observed increased number of 5S rDNA signals and their expansion on nearly all chromosomes except for the smallest chromosome pair. Increased number of signals (to as many as 16 on each of 8 chromosomal pairs) was also observed in *T. microcephalum* from the section *Involucrarium*. Joining the positions of 5S and 26S rDNA was characteristic for the species analysed from this section.

Existence of increased number of rDNA signals and their expansion on nearly all chromosomes has been described in *Canavalia gladiata*, belonging to the family Fabaceae and with 20 5S rDNA loci dispersed on 20 of its 22 chromosomes [42], as well as in *Cucumis sativus*, where 10 signals of 26S rDNA loci were dispersed on 10 of its 14 chromosomes [43]. Physical mapping of the rDNA sequences in these species revealed a significant remodelling of the 35S and 5S rDNA sites that contributed greatly to differentiation of the 2n = 16 and 2n = 12 karyotypes.

Ansari et al. reported that species of the section *Trifoliastrum* showed a unique x = 8, and they observed two rDNA hybridization patterns [37]. This coincided with our observation, as the analysed species *T. glomeratum*, *T. montanum*, and *T. thalii* showed hybridization identical (two pairs of rDNA signals on separated chromosomes) with that of *T. nigrescens* ssp. *petrisavii*, and *T. ambiguum. T. pallescens* exhibited the second pattern (co-localized 26S and 5S signals on one chromosome pair), and it was identical to that of six other species within this section.

Reduction of rDNA loci was observed in some species whereby one pair of signals was usually weaker than the second one. Reduction of 5S rDNA loci was observed in *T. stellatum* and *T. occidentale*. The same phenomenon was described in gymnosperms *Pinus nigra* and *P. taeda* for both rDNA loci [44,45]. The reduction of 5S rDNA loci is more common in monocots [46,47,48], but it is also observed in dicots, including the genus *Trifolium.* Odd numbers of 5S rDNA loci were observed in *T. glanduliferum* (Figure S6B and Figure 1C) and *T. alpestre* (Figure S2B and Figure 1D), with 5 and 11 loci, respectively. Odd numbers of 5S rDNA loci have been observed also in *Byblis*, *Tanacetum*, and *Rosa* species [49,50,51], as have odd numbers of 35S rDNA loci in *Fragaria* [52].

Polyploid *T. lupinaster* (2n = 4× = 28, 32) from the section *Lupinaster* (Figure S6E,F and Figure 1B) and *T. repens* (2n = 4× = 32) from the section *Trifoliastrum* had a similar pattern of rDNA distribution on chromosomes. In *T. repens*, Ansari et al. [37] described one 26S and two 5S sites per monoploid genome, one 5S site being in adjacent position with 26S and the other on a separate chromosome in a pericentromeric region. We, however, observed the separate 5S signal in the subterminal region. *T. repens* is a verified allopolyploid, and therefore the described similarity could justify hypothesizing an allopolyploid origin of *T. lupinaster*. The different number of 5S rDNA signals possibly points to a decreasing loci number (rDNA inactivation and loss), quite common in natural allopolyploids and which had arisen after hybridization. The same phenomenon was described by Dluhošová et al. [18] in octoploid and presumed allopolyploid *T.* *medium* (2n = 8× = 64), where 8 clusters of 35S rDNA and 12 clusters of 5S rDNA were described on separate chromosomes. The effect of polyploidy on the number and position of rDNA sites on intra- and interspecific levels has also been evaluated [25,53]. In polyploids, there is a trend towards reduction in the number of sites per monoploid complement [25,54] caused by epigenetically regulated inactivation, asymmetrical reduction of loci via unequal recombination, and loss [55]. In another allotetraploid species from the subgenus *Chronosemium*, FISH analysis of *T. dubium*, having *T. campestre* and *T. micranthum* as ancestors, revealed two pairs of each rDNA signal on different chromosomes. Ansari et al. [34] concluded that one pair of NOR in tetraploid *T. dubium* was inactivated after speciation.

Dluhošová et al. [38] described great variability by FISH using rDNA probes within the artificial interspecific hybrid *T. pratense* × *T. medium*. Individual plants had a pattern of 5S and 35S rDNA loci rather more similar to that of *T. pratense* than of *T. medium*. Numbers of chromosomes with clusters of 5S rDNA ranged from 6 to 14 while those with clusters of 35S rDNA varied between 4 and 13. Individual arrangements were almost unique, and some plants also possessed novel formations not present in either of the parental species. This suggests complex rearrangements connected with post-hybridization stabilization of hybrid genomes.

Sato et al. [27] and Dluhošová et al. [38] described the same rDNA pattern in diploid (2n = 14) and artificial autotetraploid (2n = 4× = 28) genotypes of *T. pratense*, but with exactly twice as many signals in autotetraploids. An additive pattern of sixteen 26S and sixteen 5S signals located separately on different chromosomes and detected in *T. pannonicum* (2n = 16× = 128) from the section *Trifolium* (Figure S2P) suggests that this genome is rather a natural autopolyploid than an allopolyploid.

### 3.2. Cytological and Sequence Diversity

Some of those species analysed showed population or subpopulation dispersion and diversification. In *T. subterraneum*, Falistocco et al. [10] described one pair of each 26S and 5S rDNA signal on different chromosome pairs. One additional pair of 26S signal was observed in our study (Figure S2Q). Dissimilarities were also observed by Ansari et al. [36]. They described separated 5S rDNA signal rather proximally in *T. occidentale* and one additional 5S rDNA signal in *T. repens*. In another five species across the whole genus—*T. badium* (subgenus *Chronosemium*), *T. glanduliferum* (section *Paramesus*), *T. microcephalum* (section *Involucrarium*), *T. spumosum* (section *Vesicastrum*), and *T. alpestre* (section *Trifolium*)—different numbers and localizations of 5S and 26S rDNA were observed. The most prominent distinction was observed in *T. microcephalum* (Figure S5C,D and Figure 1D), where 26S and 5S signals co-localized on all chromosomes or 26S and 5S signals co-localized on one pair of chromosomes with 5S signals on all remaining chromosomes. A range of variants occurring within a single species can be the main force driving diversity not only in chromosome numbers but also in rDNA loci count and localization within that species across fields and countries. These intraspecies and interpopulation differences exist also elsewhere within the plant kingdom [56], specifically in the genus *Anacyclus* belonging to the Asteraceae family. Similarly, *Medicago truncatula* lines Jemalong J5 and R-108-1 exhibited differences in location of the 5S rDNA [29].

Sequence alignment of the 85 analysed *Trifolium* sequences revealed correlation between mean 26S rDNA number per chromosome and polymorphism content in ITS1 sequence. Three kinds of sequence variation (single nucleotide variation, indel, and larger deletion) were quantified and reduced diversity was observed in ITS1 sequences in *Trifolium* species with no significantly lower GC content in three regions (ITS1, 5.8S, ITS2). This suggests an absence of pseudogenes. Higher ITS sequence diversity in polyploids has often been described and then attributed to frequent interspecies hybridization events [57,58,59]. In our study, ITS1 diversity with respect to single nucleotide variations was higher both in polyploids and diploids with reduced basic chromosome numbers than in diploids with ancestral basic chromosome number, and indel diversity was greatest in diploids with reduced basic chromosome numbers. Diversity in diploids with reduced basic chromosome numbers could have arisen from differential accumulation of repetitive sequences [59].

Our results contribute to improved understanding of the *Trifolium* genome structure and evolutionary history. Further studies addressing the mechanism of chromosome number reduction, comparative analyses with evolutionarily close legumes, or the identification of parental origin for many allopolyploid species have yet to be conducted. Expanding cytogenetic and cytogenomic research in Fabaceae and the *Trifolium* genus using such advanced methods as oligonucleotide libraries or bacterial artificial chromosome libraries (oligo-FISH/-painting, BAC-FISH/-painting) will expand our understanding of genome organization [60,61,62,63], structural rearrangements [64], and the genome dynamics in allopolyploids [65,66].

## 4. Materials and Methods

### 4.1. Plant Material and Chromosome Preparation

Table 2 lists the 30 *Trifolium* species investigated here and their seed origins. Seeds were germinated on Petri dishes at 8–10 °C for 24–48 h, then transferred at 23 °C. Seedlings were placed in pots with perlite supplemented with Murashige and Skoog medium [67]. Actively growing root tips were collected either from germinated seeds or from growing plants.

The entire protocol of preparing root tips and the slides for FISH followed protocols by Lysák and Mandáková [68] and Kirov et al. [69] with little modification. Root tips were pretreated in cold water overnight and fixed in freshly prepared 100% ethanol and 99% acetic acid in a 3:1 ratio. Root tips were washed in a 0.1 M citrate buffer (0.08 M sodium citrate dihydrate, 0.01 M citric acid) and digested in 30 µL of enzymatic mixture containing 0.3% cellulase, 0.3% pectolyase, and 0.3% cytohelicase (Merck, Prague, Czech Republic) in a citrate buffer for 80–100 min at 37 °C. The cell suspension was vortexed and 470 µL of water was added. It was then centrifuged at 10,000 rpm (Eppendorf 5415C centrifuge) for 2 min. Supernatant was removed, 470 µL of 100% ethanol was added, and the mixture was again centrifuged at 11,000 rpm for 2 min. The pellet was resuspended in ethanol (10 µL per slide). Ten microlitres of suspension was dropped onto a slide and 20 µL of the first fixation (3:1 ethanol and acetic acid) was applied. This slide was held upside down over the steam from a water bath at 55–60 °C for 15–20 s, then 5 µL of the second fixation (2:1 ethanol and acetic acid) was added and the previous step repeated. The slide was then dried at room temperature and stored in a refrigerator at 8–10 °C. The slides with chromosome spreads were treated with 100 µg mL^−1^ RNase A (Sigma, St. Louis, MO, USA) in saline–sodium citrate buffer (2× SSC; 0.3 M sodium chloride, 30 mM trisodium citrate, pH 7.0) for 1 h at 37 °C, then with 0.1 µg mL^−1^ pepsin in 10 mM HCl for 5 min at 37 °C, and finally washed two times in 2× SSC and a 70–90–100% ethanol series.

### 4.2. Probe DNA Isolation and Labelling

To localize the clusters of 26S rDNA and 5S rDNA, sequence lengths of 899 bp from *Arabidopsis thaliana* (L.) Heynh. (X52320.1, GenBank) and 117 bp from *T. repens* (AF072692.1, GenBank), respectively, were used for primers design. Selected rDNA sequences were amplified using specific primers (26S_F: TTCCCACTGTCCCTGTCTACTAT, 26S_R: GAACGGACTTAGCCAACGACA; 5S_F: GGTGCGATCATACCAGCACTAA, 5S_R: GAGGTGCAACACAAGGACTTC) by polymerase chain reaction (PCR). The PCR mixture contained: 1× GoTaq Reaction Buffer (Promega, Madison, WI, USA), 0.2 mM dNTPs, 1 μL primers, 0.5 U Taq Polymerase (Promega, Prague, Czech Republic), and 20 ng of gDNA (*T. pratense* var. Tatra). PCR products were separated by electrophoresis in 3% agarose gel, excised from the gel, purified using a PCR extraction kit (Qiagen, Hilden, Germany), then quantified using a NanoDrop 2000c spectrophotometer (Thermo Scientific). Probes were labelled by nick translation using biotin and digoxigenin Nick Translation Mix (Roche, Mannheim, Germany).

### 4.3. Fluorescence In Situ Hybridization

A denaturation mixture in volume 25 µL containing 12.5 µL 100% formamide, 5 µL 50% dextran sulphate, 2.5 µL 20× SSC, 3 µL water, and with 1 µL of each probe (26S, 5S) in a final concentration of 100 ng per used volume was denatured at 96 °C for 10 min, then rapidly cooled for 2 min. The mixture was applied onto a chosen slide and co-denatured on a hot plate at 80 °C for 2 min, then incubated overnight at 37 °C in a humid chamber box. Post-hybridization washing was carried out at 42 °C with the following steps: 2× SSC twice for 5 min, 10% formamide in 0.1× SSC twice for 5 min, 2× SSC for 5 min, and 4× SSC with 0.05% Tween-20 for 5 min. Biotin and digoxigenin-labelled probes were immunodetected using streptavidin-Cy3 (GE Healthcare, Buckinghamshire, United Kingdom; 1:750 dilution) and anti-DIG-FITC (Roche; 1:250 dilution) antibodies, respectively. Chromosomes were counterstained with 4′,6-diamidino-2-phenylindole (DAPI) in Vectashield (Vector Laboratories, Burlingame, CA, USA).

Images were captured using an Olympus BX 51, Olympus, Tokyo, Japan fluorescence microscope equipped with an Olympus DP72 CCD camera. Three greyscale images of each mitosis event were taken, and images were pseudocoloured using Adobe Photoshop CS6 software (chromosomes—blue, 26S rDNA signals—green, 5S rDNA signals—red).

### 4.4. Phylogenetic Tree Construction

ITS1 *Trifolium* sequences of 42 species with known rDNA loci numbers were used for tree construction with *A. thaliana* ITS1 sequence taken as outgroup. These sequences were obtained from the National Center for Biotechnology Information (NCBI) GenBank (Table S1). Sequences in FASTA format were aligned by Clustal W type in MEGA-X and saved in NEXUS format. The data set was converted to xml format in BEAUTi version 1.10.4. Bayesian analysis of sequences was done in BEAST version 1.10.4, the final file was cleaned according to conventional protocol procedure in Tree Annotator 1.10.4, and posterior probability was fixed at >95. The phylogeny tree was visualized in Mesquite (v.3.04) and *A. thaliana* was omitted from the final visualization. The model for reconstructing ancestral state was implemented in Mesquite (v.3.04) while using that software’s “parsimony” method. Coloured elements and labels were added in Adobe Photoshop CS6.

### 4.5. Identification of Specific Polymorphisms

To identify polymorphisms specific for individual sections of the *Trifolium* genus, 85 nucleotide sequences were obtained from NCBI GenBank (Table S2). Sequences were aligned using the MUSCLE algorithm implemented in MegAlign Pro of the DNASTAR software package (DNASTAR, Madison, WI, USA), and polymorphisms were identified by grouping the aligned sequences under the sections. Specificity of the polymorphisms was assessed using Fisher’s exact test in R (ver. 3.6.1), and polymorphisms with *p*-value < 0.05 were considered as significant. Genetic diversity of the ITS1 sequence was determined using DnaSP [70]. Control for the presence of pseudogenes was performed in the accessions from Table S2, which, together with ITS1 sequence, contained 5.8S region and ITS2 sequence. GC content was calculated using MEGA-X [71], and 5.8S regions were inspected for presence of the three angiosperm-conserved motifs M1 (5′-CGATGAAGAACGTAGC), M2 (5′-GAATTGCAGAATCC-3′), and M3 (5′-TTTGAACGCA-3′) while following Harpke and Peterson [72]. Secondary structures of 5.8S region were predicted using Mfold version 2.3 on a web server [73,74] under specific settings for a base pairing following Hřibová et al. [75] for helix B4, F 36 99 3; helix B5, F 42 55 3; helix B6, F65 90 3; helix B7, F 104 112 3; and helix B8, F 113 136 4 and F120 129 3.

*Trifolium* species were divided into three groups according to their ploidy and basic chromosome numbers (polyploid, diploid with ancestral basic chromosome number, and diploid with reduced basic chromosome numbers). For species with variable number of chromosomes, both alternative chromosome number (polyploidy, diploidy with reduced basic chromosome numbers) were taken into account. Genetic diversity of the ITS1 sequences was calculated using DnaSP [70]. The variability of ITS1 sequences was evaluated for SNP polymorphism and short insertion/deletion events (indels) (option: Multiallelic) in each group separately.

## 5. Conclusions

The *Trifolium* ancestral karyotype contains a single pair of 5S and 26S rDNA sites. Diversification of 5S rDNA numbers in the genus *Trifolium* occurred independently in all eight analysed sections, the earliest event having occurred in the section *Trifolium*. Diversification had the character of expanding from ancestral 1 to 2 or rarely to 3, 5, or 8 5S rDNA sites per haploid genome. Diversification of 26S rDNA sites was rarely observed. Most commonly, expansion from one to two 26S rDNA sites was observed. Increased number of rDNA signals was characteristic for diploid species with reduced basic chromosome numbers. SNP and indel polymorphisms were greater in these species in comparison with diploids with x = 8 and polyploids, which could be attributed to the evolutionary benefit.

## Figures and Tables

**Table 1:** ITS1 sequence diversity based upon 85 sequences of *Trifolium* species.

	SNP Polymorphism		Indel Polymorphism	
Diploid (ancestral basic chromosome number)	k	11.811	k(i)	1.570
	Pi	0.0642	Pi(i)	0.0063
Diploid (reduced basic chromosome numbers)	k	17.464	k(i)	4.307
	Pi	0.0939	Pi(i)	0.0176
Polyploid	k	19.864	k(i)	1.303
	Pi	0.0867	Pi(i)	0.0055

k—mean number of nucleotide differences, Pi—nucleotide diversity, k(i)—indel diversity, Pi(i)—indel diversity per site, indel—insertion/deletion.

**Table 2:** Origin and accession numbers of analysed *Trifolium* species.

Subgenus/ Section	*Trifolium* Species	Accession Number	Gene Bank	Subgenus/ Section	*Trifolium* Species	Accession Number	Gene Bank
**Chronosemium**				**Trichocephalum**	*T. subterraneum* subsp. *subterraneum* L.	TRIF259	G-DE
	*T. aureum* L.	13T0500014	PR-CZ	**Vesicastrum**	*T. fragiferum* L.	TRIF1140	G-DE
	*T. badium* Schreb.	AZ4518, AZ159	AR-NZ		*T. resupinatum* L.	TRIF1134	G-DE
**Trifolium**					*T. spumosum* L.	AZ198, 13T0500086	AR-NZ, PR-CZ
**Trifolium**	*T. alpestre* L.	TRIF210	G-DE	**Trifoliastrum**	*T. glomeratum* L.	TRIF136, TRIF142	G-DE
	*T. arvense* L.	13T0500032	PR-CZ		*T. montanum* L.	TRIF152	G-DE
	*T. bocconei* Savi.	TRIF81, TRIF93, TRIF40	G-DE		*T. occidentale* Coombe	OCD1210, OCD50	AR-NZ
	*T. cherleri* L.	TRIF135	G-DE		*T. pallescens* Schreb.	AZ6429	AR-NZ
	*T. diffusum* Ehrh.	TRIF250	G-DE				
	*T. hirtum* All.	AZ6762, TRIF213	AR-NZ, G-DE		*T. thalii* Vill.	AZ6833	AR-NZ
	*T. ligusticum* Balb. Ex Loisel	TRIF137	G-DE	**Involucrarium**	*T. chilense* Hook. & Arn.	AZ1759	AR-NZ
	*T. pallidum* Waldst&Kit	TRIF253	G-DE		*T. microdon* Hook. & Arn.	AZ6256	AR-NZ
	*T. purpureum* Loisel.	TRIF143	G-DE		*T. microcephalum* Pursh.	TRIF244	G-DE
	*T. rubens* L.	TRIF33, TRIF211	G-DE	**Paramesus**	*T. glanduliferum* Boiss.	AZ6880	AR-NZ
	*T. squamosum* L.	TRIF68	G-DE		*T. strictum* L.	TRIF109	G-DE
	*T. stellatum* L.	TRIF252	G-DE	**Lupinaster**	*T. lupinaster* L.	TRIF272	G-DE
	*T. pannonicum* Jacq.	TRIF8	G-DE				

PR-CZ (Prague Ruzyně, Czech Republic), AR-NZ (AgResearch New Zealand), G-DE (Gatersleben, Germany).

## Data Availability

All data generated or analyzed during this study are included in this published article. Further inquiries can be addressed to the corresponding author.

## Supplementary Materials

The following are available online at https://www.mdpi.com/article/10.3390/plants10091771/s1. Figure S1: Hybridization patterns of 5S and 26S rDNA FISH probes in subgenus *Chronosemium*. Figure S2: Hybridization patterns of 5S and 26S rDNA FISH probes in subgenus *Trifolium,* sections *Trifolium* (A–P) and *Trichocephalum* (Q). Figure S3: Hybridization patterns of 5S and 26S rDNA FISH probes in section *Vesicastrum*. Figure S4: Hybridization patterns of 5S and 26S rDNA FISH probes in section *Trifoliastrum*. Figure S5: Hybridization patterns of 5S and 26S rDNA FISH probes in section *Involucrarium*. Figure S6: Hybridization patterns of 5S and 26S rDNA FISH probes in sections *Paramesus* (A–D) and *Lupinaster* (E,F). Table S1: 5S and 26S rDNA loci numbers and chromosomal positions in 42 *Trifolium* species belonging to subgenus *Chronosemium* and subgenus *Trifolium* (7 Sections). Table S2: *Trifolium* accessions for analysis of ITS1 sequences. Table S3: *Trifolium* section-specific polymorphisms in ITS1 sequence. Table S4: Presence of pseudogenes in the *Trifolium* accessions from Table S2.
