# Supplementary material for: Variation in Ribosomal DNA in the Genus Trifolium (Fabaceae)
Source: Plants (Basel). 2021 Aug 25;10(9):1771. doi: 10.3390/plants10091771 (PMC8465422; doi:10.3390/plants10091771)
Supplement: Supplementary file 1 [file plants-10-01771-s001.zip › plants-1354524-supplementary/Figure S2 Trifolium+Trichocephalum.pdf]

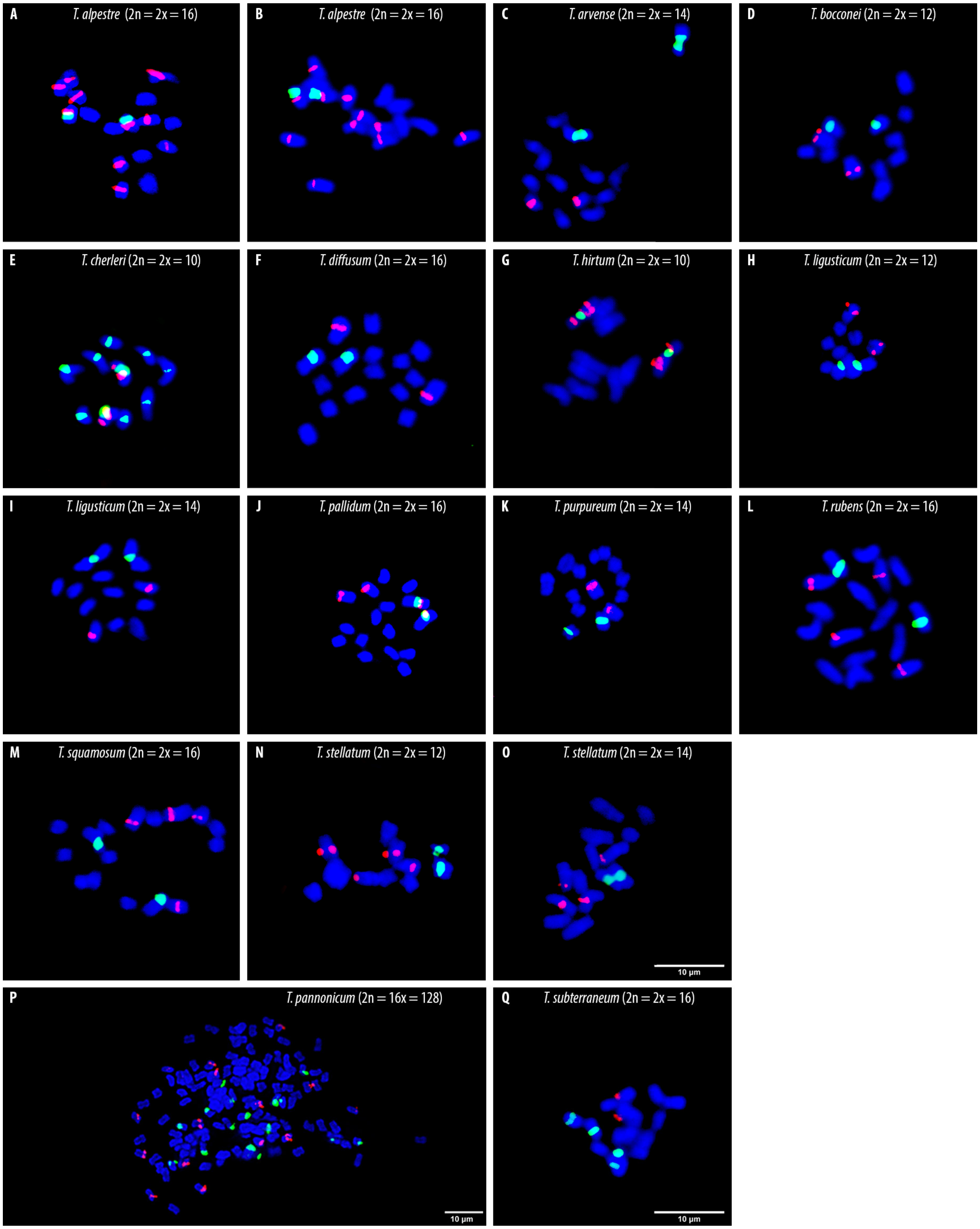

Figure S2. Hybridization patterns of 5S and 26S rDNA FISH probes in subgenus *Trifolium,* sections *Trifolium* (**A**–**P**) and *Trichocephalum* (**Q**).
