# Supplementary material for: Variation in Ribosomal DNA in the Genus Trifolium (Fabaceae)
Source: Plants (Basel). 2021 Aug 25;10(9):1771. doi: 10.3390/plants10091771 (PMC8465422; doi:10.3390/plants10091771)
Supplement: Supplementary file 1 [file plants-10-01771-s001.zip › plants-1354524-supplementary/Figure S6 Lupinaster+Paramesus.pdf]

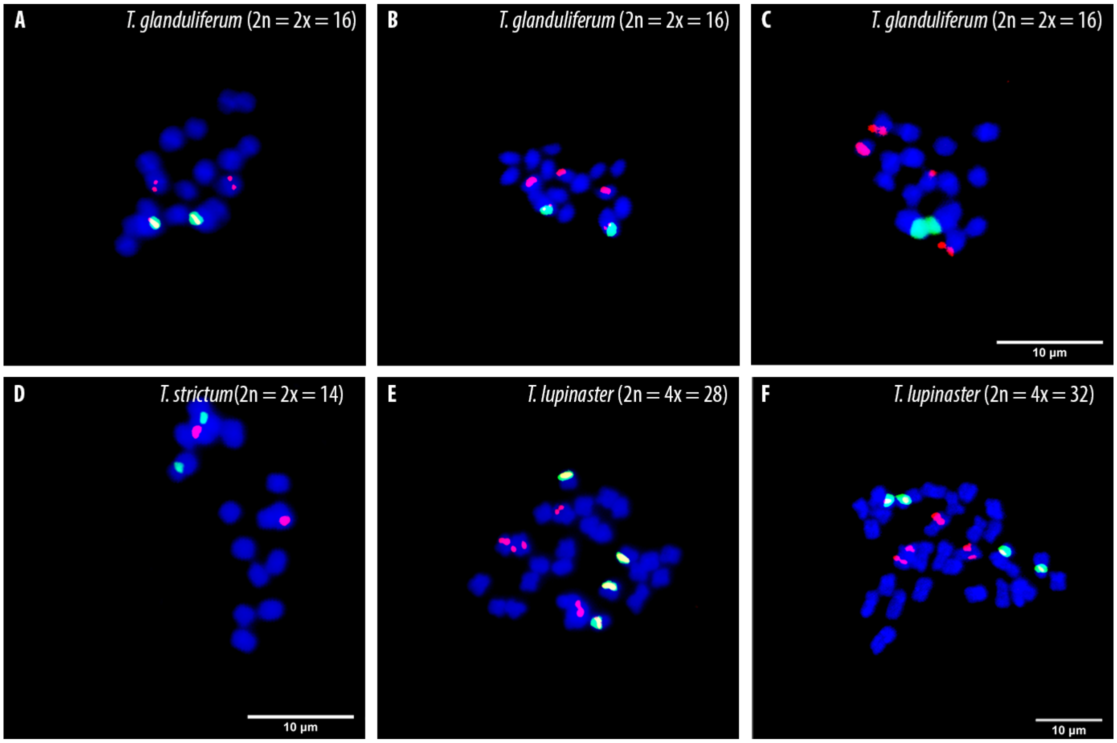

Figure S6. Hybridization patterns of 5S and 26S rDNA FISH probes in sections *Paramesus* (**A**–**D**) and *Lupinaster* (**E**,**F**). .
