# Supplementary material for: Variation in Ribosomal DNA in the Genus Trifolium (Fabaceae)
Source: Plants (Basel). 2021 Aug 25;10(9):1771. doi: 10.3390/plants10091771 (PMC8465422; doi:10.3390/plants10091771)
Supplement: Supplementary file 1 [file plants-10-01771-s001.zip › plants-1354524-supplementary/Table S1.pdf]

Table S1. 5S and 26S rDNA loci numbers and chromosomal positions in 42 *Trifolium* species belonging to subgenus *Chronosemium* and subgenus *Trifolium* (7 sections).

**Subgenus/Section**	***Trifolium* species**	**2n**	**Loci number per 2n**		**Position**
			5S	26S	
**Chronosemium**					
	T. aureum ▪	2x=16	4	2	AS OCP
		2x=14	4	2	AS OCP
	T. badium ▪	2x=14	2	4	S
			2	2	S
	T. campestre [a] ▪	2x=14	2	2	S
	T. micranthum [a] ▪	2x=16	2	2	S
	T. dubium [a] ▪	4x=30	4	4	S
**Trifolium**					
Trifolium	*T. alpestre* ▪	2x=16	10	2	CBS1 AS
			11	2	CBS1 AS
	T. arvense ▪	2x=14	2	2	S
	T. bocconei ▪	2x=12	2	2	S
	T. cherleri ▪	2x=10	4	10	CBS1 AS
	T. diffusum ▪	2x=16	2	2	S
	T. hirtum ▪	2x=10	6	2	AS OCP
	T. ligusticum ▪	2x=12	2	2	S
		2x=14	2	2	S
	T. pallidum ▪	2x=16	4	2	CBS1 AS
	T. purpureum ▪	2x=14	2	2	S
	T. rubens ▪	2x=16	4	2	S
	T. squamosum ▪	2x=16	4	2	S
	T. stellatum ▪	2x=12	4	2	S
		2x=14*	4 (2w)	2	S
	T. pannonicum ▪	16x=128	16	16	S
	T. pratense [b] ▪	4x=28	8	8	CBS2 AS
	T. medium [b] ▪	8x=64	12	8	S
Trichocephalum	*T. subterraneum* subsp. *subterraneum* ▪	2x=16	2	4 (2w)	S
	T. subterraneum subsp. *subterraneum* [c] ▪	2x=16	2	2	S
	T. subterraneum subsp. *brachycalycinum* [c]	2x=16	2	4 (2w)	S
	T. israeliticum [c] ▪	2x=12	10	4	CBS2 AS
Vesicastrum	*T. fragiferum* ▪	2x=16	2	2	AS OCP
	T. resupinatum ▪	2x=16	2	2	AS OCP
		2x=14	2	2	AS OCP
	T. spumosum ▪	2x=16	2	2	S
			4	2	CBS1 AS
Trifoliastrum	*T. glomeratum* ▪	2x=16	2	2	S
	T. montanum ▪	2x=16	2	2	S

	T. occidentale [d] ▪	2x=16	4	2	CBS1 AS
	T. pallescens ▪	2x=16	2	2	AS OCP
	T. thalii ▪	2x=16	2	2	S
	T. repens [d] ▪	4x=32	4	2	CBS1 AS
	T. uniflorum [d] ▪	4x=32	4	4	CBS2
	T. nigrescens subsp. *nigrescens* [d]	2x=16	2	2	CBS1
	T. nigrescens subsp. *petrisavii* [d] ▪	2x=16	2	2	S
	T. ambiguum [d] ▪	2x=16	2	2	S
	T. hybridum [d] ▪	2x=16	2	2	CBS1
	T. isthmocarpum [d]▪	2x=16	2	6	CBS1 AS
Involucrarium	*T. chilense* ▪	2x=16	4	2	CBS1 AS
	T. microdon ▪	2x=16	2	2	CBS1
	T. microcephalum ▪	2x=16	16	16	CBS8
			16	2	CBS1 AS
Paramesus	*T. glanduliferum* ▪	2x=16	4	2	CBS1 AS
			5 (1w)	2	CBS1 AS
			4	2	S
	T. strictum ▪	2x=14	2	2	S
Lupinaster	*T. lupinaster* ▪*	4x=28*	8	4	CBS2 AS
		4x=32	8	4	CBS2 AS

* Newly described chromosome number. Published in [a]Ansari et al. [33], [b]Dluhošová et al. [37], [c]Falistocco et al. [13], [d]Ansari et al. [36]. w – weaker signals than the others; S – signals on separate chromosomes; AS OCP – all signals on one chromosome pair; CBSn AS – co-localization of both signals on n-chromosome pairs, another on separate chromosomes; CBSn – co-localization of both signals on n-chromosome pairs. ▪ indicates sequences used in the tree (Figure S7), with *A. thaliana* used as an outgroup.
