# Supplementary figures and images for: Variation in Ribosomal DNA in the Genus Trifolium (Fabaceae)
Source: Plants (Basel). 2021 Aug 25;10(9):1771. doi: 10.3390/plants10091771 (PMC8465422; doi:10.3390/plants10091771)

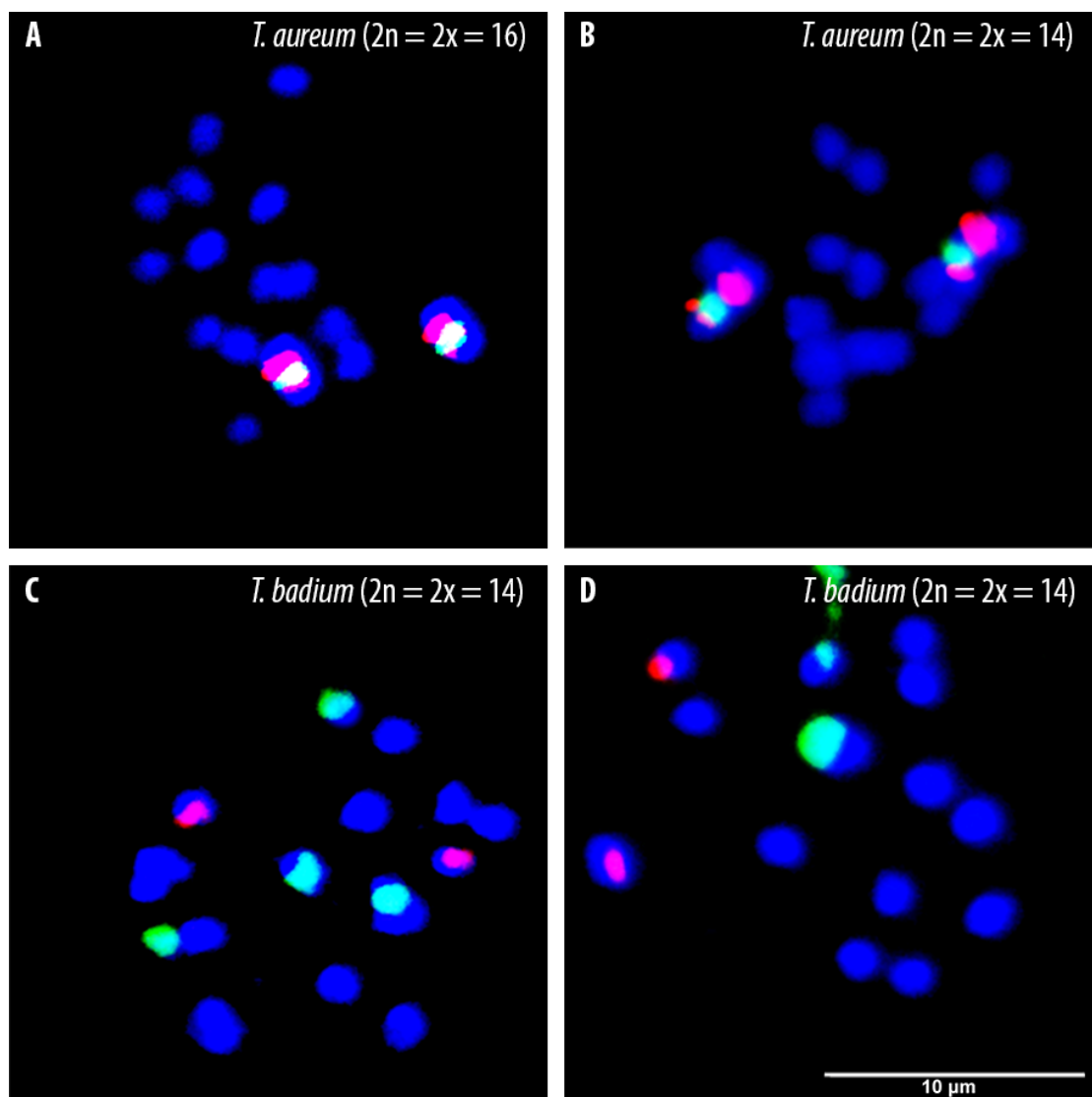

**Figure S1.** Hybridization patterns of 5S and 26S rDNA FISH probes in subgenus *Chronosemium*.

Supplement: Supplementary file 1 [file plants-10-01771-s001.zip › plants-1354524-supplementary/Figure S1 Chronosemium.pdf]

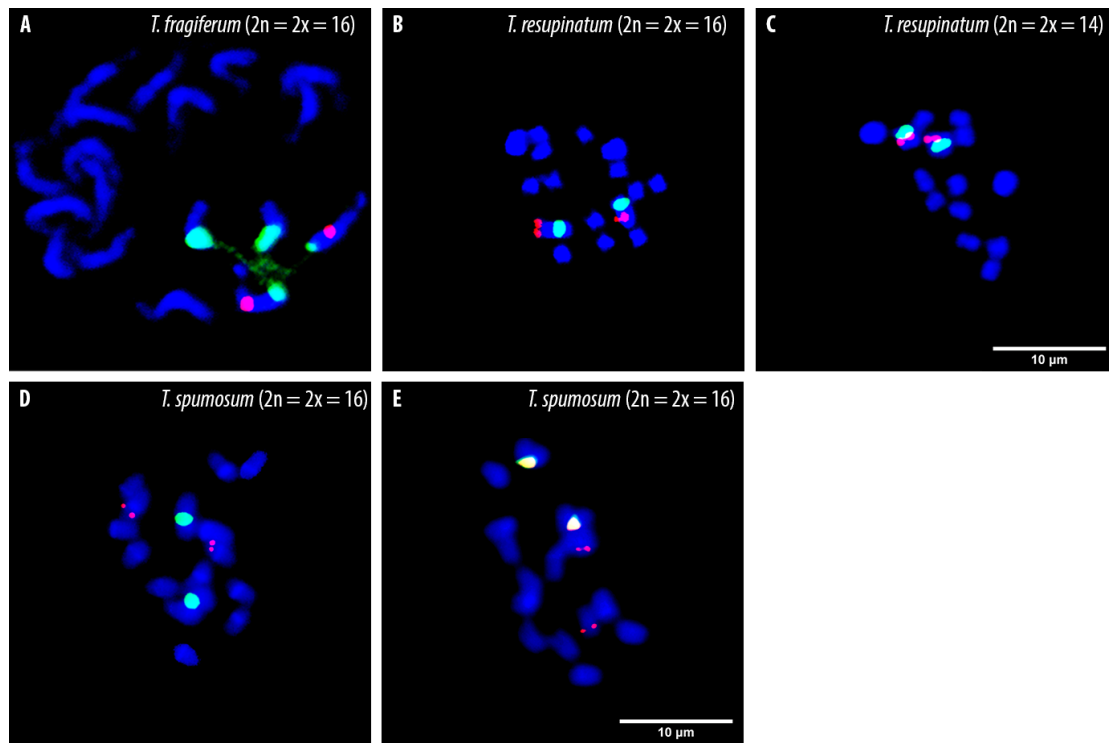

**Figure S3.** Hybridization patterns of 5S and 26S rDNA FISH probes in section *Vesicastrum*.

Supplement: Supplementary file 1 [file plants-10-01771-s001.zip › plants-1354524-supplementary/Figure S3 Vesicastrum.pdf]

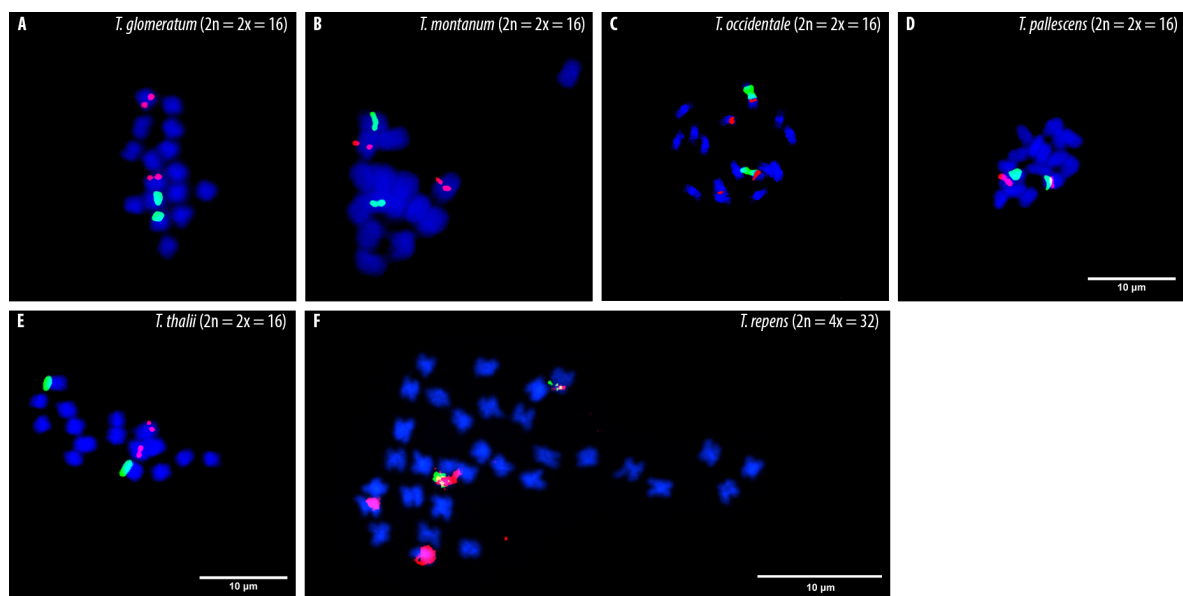

**Figure S4.** Hybridization patterns of 5S and 26S rDNA FISH probes in section *Trifoliastrum*.

Supplement: Supplementary file 1 [file plants-10-01771-s001.zip › plants-1354524-supplementary/Figure S4 Trifoliastrumc.pdf]

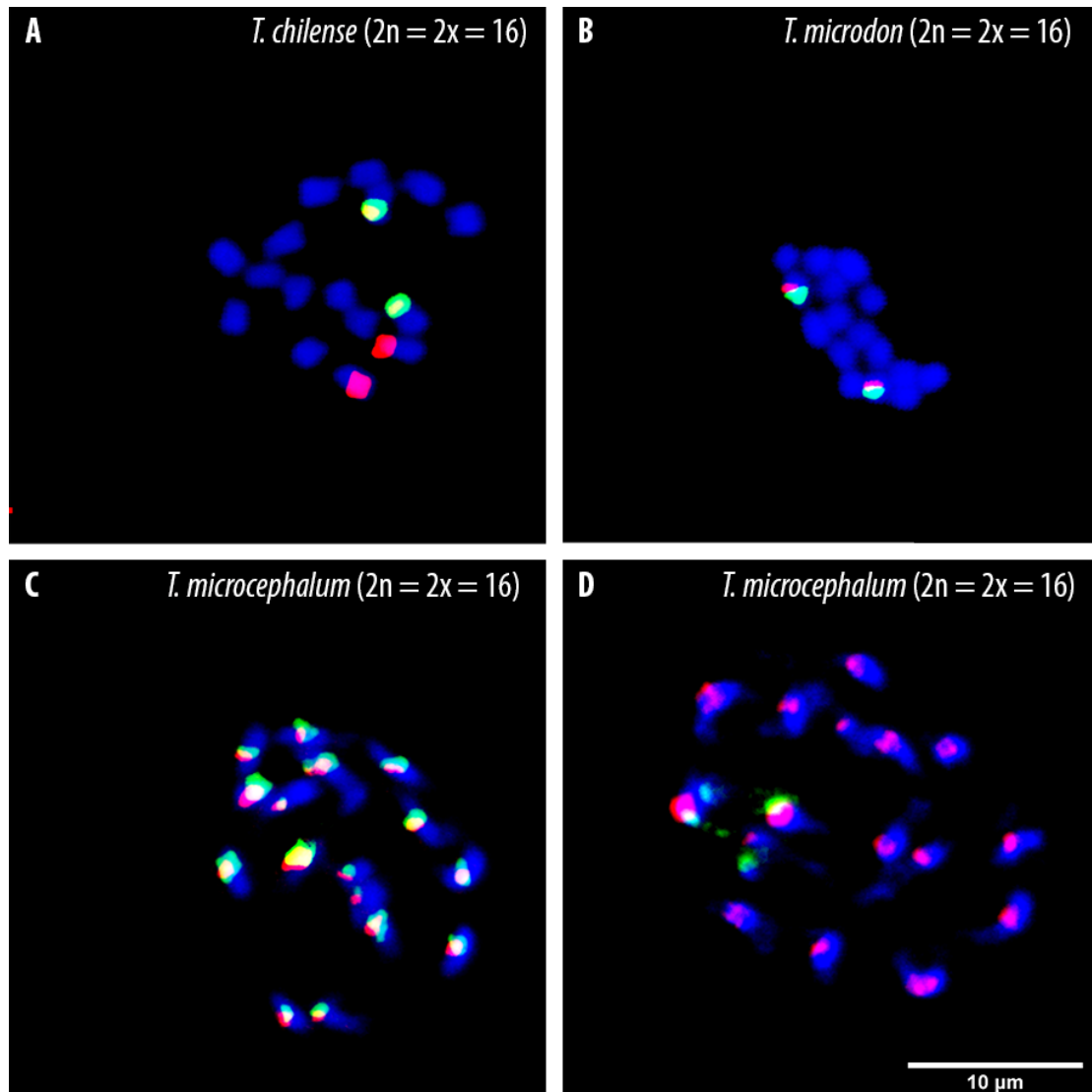

**Figure S5.** Hybridization patterns of 5S and 26S rDNA FISH probes in section *Involucrarium*. .

Supplement: Supplementary file 1 [file plants-10-01771-s001.zip › plants-1354524-supplementary/Figure S5 Involucrarium.pdf]
